# Neonatal hemolytic anemia does not always indicate thalassemia: a case report

**DOI:** 10.1186/s13104-017-2803-6

**Published:** 2017-09-12

**Authors:** Arwa A. Al-Harazi, Bilguis M. Al-Eryani, Butheinah A. Al-Sharafi

**Affiliations:** 1 Department of Pediatrics, School of Medicine and Health Sciences, Sana’a University, PO Box 700, Sana’a, Yemen; 2 Department of Medicine, School of Medicine and Health Sciences, Sana’a University, Sana’a, Yemen

**Keywords:** Neonatal, Congenital erythropoietic porphyria, Alpha thalassemia, Case report, Hemolytic anemia

## Abstract

**Background:**

Congenital erythropoietic porphyria is a rare autosomal recessive disorder that affects heme-porphyrin synthesis. This disorder is due to the genetic defect of uroporphyrinogen III cosynthase. This defect results in the accumulation of high amounts of uroporphyrin I in all tissues, leading to clinical manifestations ranging from mild to severe chronic damage of the skin, cartilage and bone. Hypertrichosis, erythrodontia and reddish-colored urine are often present, as well as hemolytic anemia accompanied by hepatosplenomegaly.

**Case presentation:**

Here, we present a case of a 5-year-old male child of Middle Eastern origin who had been diagnosed as having alpha thalassemia and was undergoing chronic blood transfusions. He later presented with hypopigmented skin lesions and atrophy post-photosensitivity, persistent red-colored urine and hepatosplenomegaly. Laboratory investigations showed a high level of porphyrin metabolites in his plasma and erythrocytes. As a result, he was diagnosed as having Congenital erythropoietic porphyria.

**Conclusion:**

Here, we diagnose a case of congenital erythropoietic porphyria which was initially missed, although the clinical features were clear (red-colored urine, hepatosplenomegaly and hemolytic anemia were present since birth, and skin manifestations appeared at the age of 22 months after being exposed to sunlight). After a DNA test was performed, the patient was initially diagnosed as having alpha thalassemia. We identified two causes of hemolytic anemia (congenital erythropoietic porphyria and alpha thalassemia) in this patient. The diagnosis of congenital erythropoietic porphyria was missed up until the child turned 5 years old. To our knowledge, this is the first case of hemolytic anemia to be reported with a diagnosis of both congenital erythropoietic porphyria and alpha thalassemia.

## Background

Congenital erythropoietic porphyria (Günther disease) (CEP) is a rare autosomal recessive disorder that affects the enzyme uroporphyrinogen III synthase. Its clinical spectrum ranges from nonimmune hydrops fetalis caused by severe hemolytic anemia in utero to late-onset mild cases of light-sensitive cutaneous lesions that result in mutilating scars in adults [1]. This enzyme defect leads to the accumulation of type I isomer porphyrins. Those are toxic, causing clinical manifestations that range from mild to severe cutaneous photosensitivity, observed starting at infancy and exhibited as mutilating skin lesions. Furthermore, erythrodontia, chronic hemolysis, splenomegaly, and massive porphyrinuria are also observed [2, 3]. The severity of the clinical manifestations is markedly heterogeneous among the patients [3]. The outlook is favorable after bone marrow or stem cell transplantation. Here, we report a case of a 5-year-old boy who was treated as having thalassemia and who presented later with hypopigmented skin lesions post-sunlight exposure, along with persistent red-colored urine and hepatosplenomegaly.

## Case presentation

A 5-year-old boy of Middle Eastern origin presented to us with skin lesions, abdominal distension and red-colored urine. He was the second child of a first-cousin consanguineous marriage. The first child had died at birth due to cord strangulation. Our patient was the product of a full-term pregnancy without antenatal care; delivery was normal, carried out at a hospital, and the infant cried immediately after birth. His birth weight was small (2200 g), and his head circumference was normal (33 cm) with a wide anterior fontanel (10 × 7 cm). His condition started at birth with poor and decreased suckling activity, reddish urine and ecchymosis all over his body. His coloration was pale with no bleeding, jaundice or fever. Physical examination at that time revealed a parasternal soft pansystolic murmur, splenomegaly located 6 cm below the costal margin (BCM) and a liver that was palpable 2 cm BCM. The differential diagnosis included hemolysis, metabolic diseases, septicemia, TORCH, congenital leukemia and mucopolysaccharidosis (MPS). An ophthalmological examination showed bilaterally sloughing corneas, dry eyes, early lens opacity and bilaterally hyperpigmented retinas. He was admitted to the hospital and was treated as a case of neonatal septicemia; he received antibiotics, multiple blood transfusion, and l-thyroxine for 2–3 months. There was no improvement in his symptoms and the family traveled abroad for further investigations. The results of the complete blood count and the thyroid function tests done at birth can be seen in Table 1.

**Table 1:** Laboratory tests done for the patient at birth

Laboratory test	Result	Normal range
Hemoglobin	6.6 g/dl	13.5–19.5 g/dl
PCV	20%	45–65%
Reticulocyte count	12%	3–7%
White blood cells	4.9 × 10^3^/mm^3^	5–20 × 10^3^/mm^3^
Neutrophil	66%	15–30–5%
Lymphocyte	21%	43–60%
Platelet count	96 × 10^3^/mm^3^	150–450 × 10^3^/mm^3^
FT4	17.5 ng⁄dl	0.7–1.7 ng⁄dl
TSH	5.8 mlU/l	0.6–6.3 mIU/l

Other tests that were conducted included the following: a blood smear, which showed normocytic normochromic anemia; a normal osmotic fragility test; and a negative Coombs test. Partial thromboplastin time (PTT), prothrombin time (PT), liver and renal function tests were also within the normal range. A TORCH screen was negative; a bone marrow aspiration was normal with no evidence of leukemia or myelofibrosis. An echocardiogram revealed a perimembranous ventricular septal defect (VSD). An abdominal ultrasound revealed huge splenomegaly with a patent portal vein. A DNA study was also done and revealed heterozygosity for alpha thalassemia, which goes along with the diagnosis of having the alpha thalassemia trait. Other tests performed ruled out Gaucher’s Disease and MPS. The patient’s ammonia and lactate levels were normal, while his serum ferritin level was elevated (449 mg/ml). The results of a skeletal survey were normal. The patient was managed with regular blood transfusions (2–3 times per month) and an iron-chelating agent (Desferrioxamine).

**Fig. 1:**
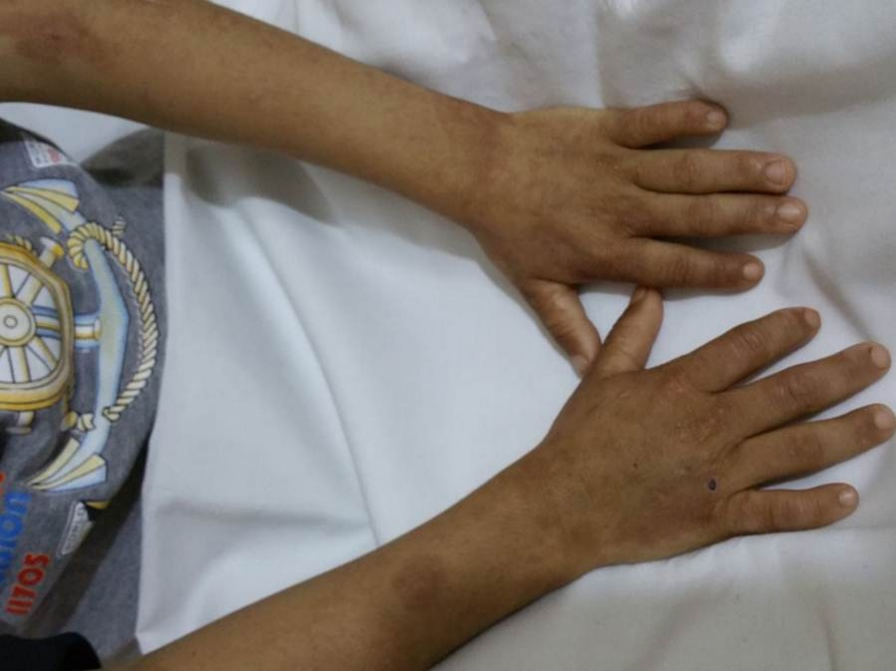
Congenital erythropoietic porphyria. Pictures of the patient’s forearms and hands are presented showing hypopigmented areas and atrophic skin lesions

**Fig. 2:**
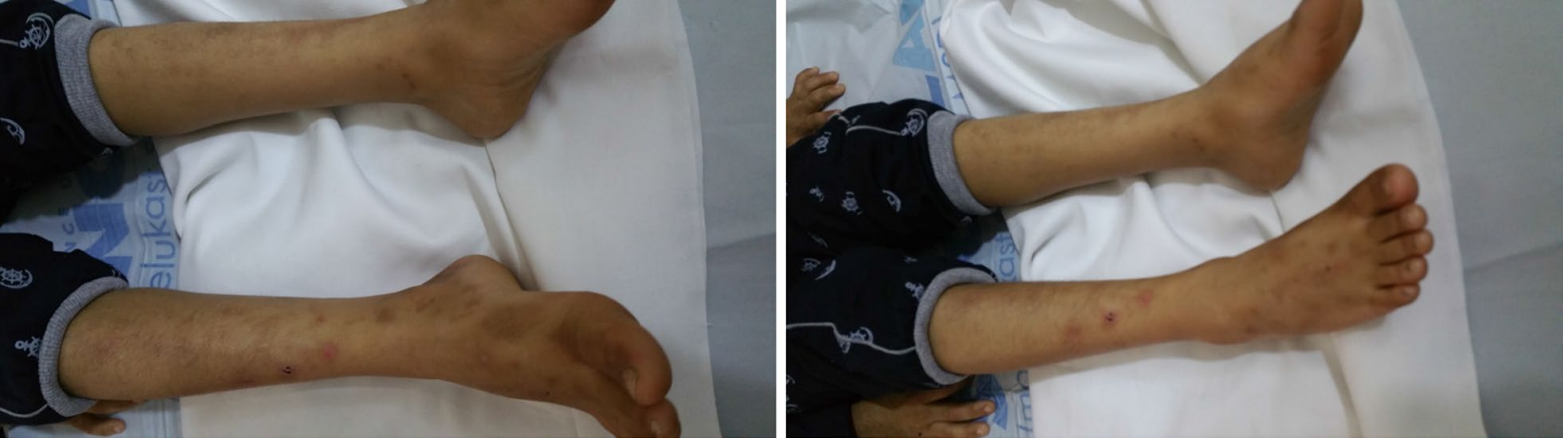
Congenital erythropoietic porphyria. Pictures shown are of lower limbs of the patient with hypopigmented areas and atrophic skin lesions. The remains of one vesiculobullous lesion can be seen

**Fig. 3:**
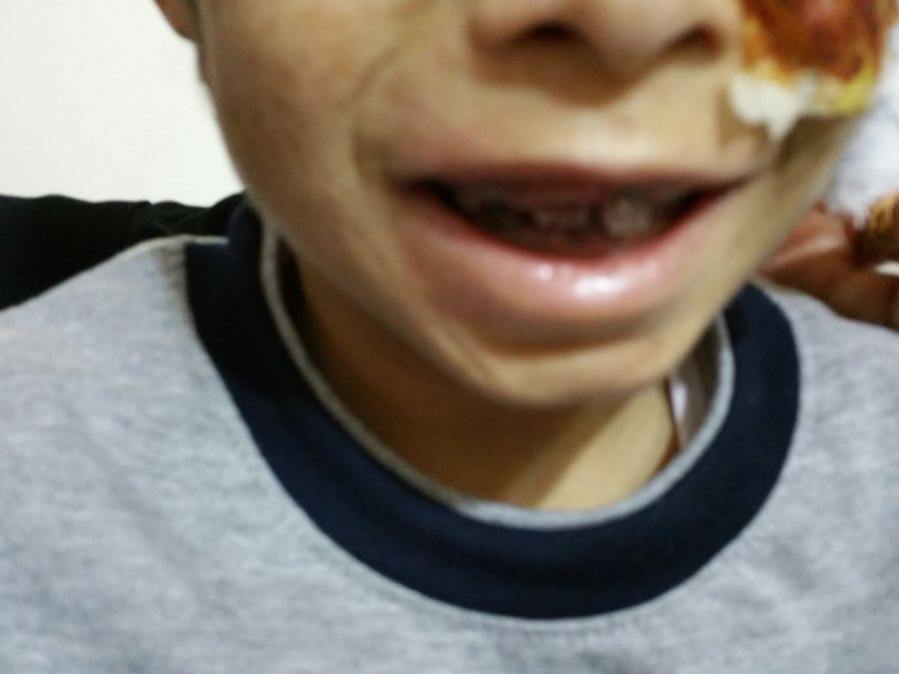
Congenital erythropoietic porphyria. Pictures of facial skin with areas of atrophy and hypopigmentation are shown. Teeth have erythrodontia

**Fig. 4:**
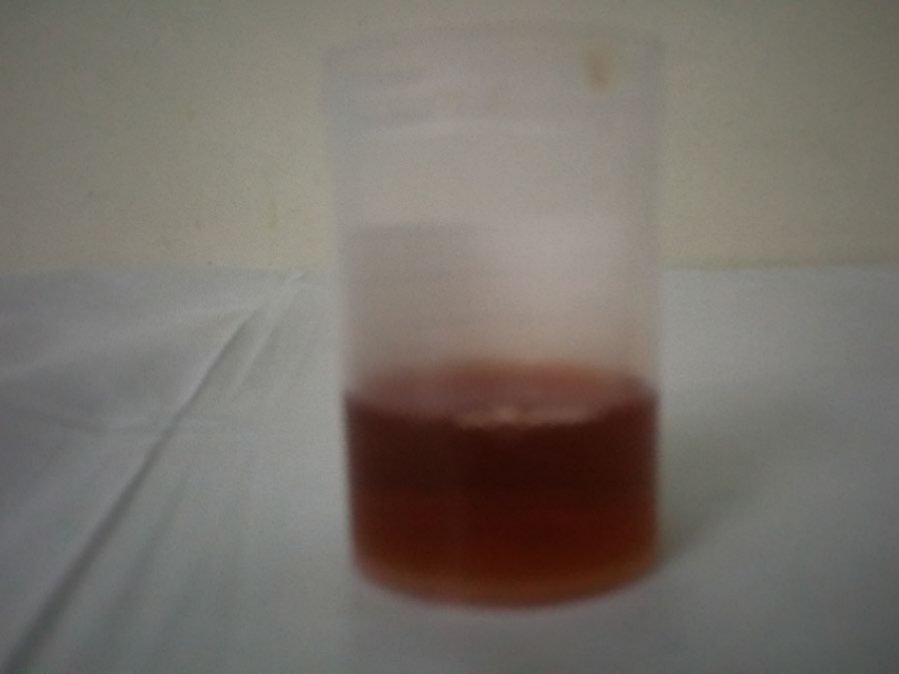
Congenital erythropoietic porphyria. Urine sample showing red colored urine

At the age of 10 months, the patient exhibited an increase in abdominal distension, with eruption of brownish-stained teeth, while continuing to have reddish urine. At the age of 22 months, he started developing vesiculobullous skin lesions in sun-exposed areas (face, hands and feet). The lesions left behind hypopigmented areas upon recovery. At 30 months of age, and due to improvement, blood transfusions were stopped, however, the skin lesions progressed (Figs. 1, 2), and the red urine (Fig. 4) and brown teeth persisted (Fig. 3). Continued investigations showed elevated serum ferritin levels, turbid urine with more than two proteins, elevated urobilinogen levels, an RBC count of 10–12 cells/HPF, and numerous WBCs. Furthermore, a culture of the urine grew *E. coli*. To rule out other hemoglobinopathies that cause hemolysis, we performed a hemoglobin electrophoresis test that showed a result of Hemoglobin AA. This meant that the patient had a normal hemoglobin electrophoresis result and a normal hemoglobin as seen in adults. At the age of 5, his condition progressed with symptoms of worsening skin lesions and persistent red urine and anemia. The patient came to our hospital for a consultation, and we diagnosed him clinically as having congenital erythropoietic porphyria. Tests were ordered to confirm this diagnosis and revealed the following results: Uroporphyrin I concentration = 28.1 mmol/ml (normal value range is 0–4); Coproporphyrin I concentration = 10.8 mmol/ml (normal value range is 0–6); Uroporphyrin I test in RBC = weakly positive; and Heptacarboxyl porphyrin levels = 33.4 (normal value range is 0–2). Although the presence of Heptacarboxyl porphyrins is normally in line with a diagnosis of hepato-erythropoietic porphyria (HEP) which has the same clinical manifestations as CEP, it can also be present in cases with CEP itself [4, 5]. We relied in our diagnosis on the clinical manifestations of the disease and on the presence of uroporphyrin I and coproporphyrin I, which occur only in CEP as a result of an absence of UROS activity. Furthermore, erythrodontia and an elevated ferritin serum levels are not features of HEP [6]. Further studies to detect the presence of mutations in the UROS and GATA1 genes are needed to confirm the diagnosis, but we were not able to perform these experiments due to the unavailability of the tests in our country.

The final diagnosis for this case was congenital erythropoietic porphyria. The mother was advised to keep the patient away from the sun in order to avoid further damage to his skin. The patient was prescribed frequent blood transfusions, and was given a regimen of desferrioxamine and vitamins.

## Discussion

Congenital erythropoietic porphyria is an extremely rare inborn defect affecting the metabolism of porphyrin heme synthesis. Only several hundred cases have been reported worldwide [7, 8], and our knowledge, this is the first case to be associated with the alpha thalassemia trait. CEP is an autosomal recessive disorder affecting the enzyme uroporphyrinogen III synthase, resulting in clinical manifestations ranging from nonimmune hydrops fetalis to a mild form of cutaneous photosensitivity lesions in adult life [9, 10]. Ocular manifestation includes blepharitis, cicatricial ectropion, conjunctivitis, and lagophthalmos with subsequent bilateral corneal scarring occurring, eventually leading to blindness [2, 11].

Alpha thalassemia is one of the world’s most common single-gene disorders and is inherited as an autosomal recessive disorder. It is commonly found in Africa, the Middle East, India, Southeast Asia, Southern China, and occasionally the Mediterranean region [12]. Alpha globin protein is made from four genes, two on each strand of chromosome 16. A person with a deletion in one of the alpha globin genes is said to be a silent carrier of α-thalassemia, which generally does not cause anemia. However, a person with a deletion in two α-globin genes on the same chromosome (*cis* position) or on each chromosome in the pair (*trans* position) is characterized as having the α-thalassemia trait. This normally causes a mild microcytic and hypochromic anemia that is often mistaken with iron deficiency anemia. Whereas deletion of three α-globin genes results in HbH disease, which is enough to cause moderate to severe anemia, as well as hepatosplenomegaly. The most severe form of the disease is that resulting from the deletion of four α-globin genes and is known as α-thalassemia major. Infants with this condition develop hydrops fetalis syndrome and usually die in utero or shortly after birth [13]. Hemoglobin electrophoresis testing is not sensitive enough to diagnose α-thalassemia, so PCR (polymerase chain reaction) and restriction endonuclease tests may be used. These tests, in addition to the DNA technology, can be diagnostic [14]. The frequency range of alpha thalassemia alleles is 5–10% in the Mediterranean basin, 20–30% in portions of West Africa, and as high as 60–80% in parts of Saudi Arabia, India, Thailand, Papua New Guinea, and Melanesia [15].

Congenital erythropoietic porphyria can resemble epidermolysis bullosa due to the occurrence of skin blistering, scarring, and mutilation. Furthermore, it is also important to exclude medication-induced pseudoporphyria, which closely resembles CEP. Elevated porphyrin levels in the urine, plasma, and stool will differentiate CEP from these other conditions. Furthermore, in other photodermatosis disorders, inflammation is not severe enough to produce cutaneous blisters [16]. The presence of erythrodontia is practically pathognomonic of CEP [17]. CEP should be considered in all cases presenting with cutaneous photosensitivity, red-colored urine and blistering of sun exposed areas [18]. As observed in our case, manifestations of CEP include bullous lesions on photosensitive exposed areas, atrophic scars and hypopigmentation, as well as erythrodontia [8]. In addition to the clinical manifestations, the diagnosis can be confirmed by observing the increase of the porphyrin fraction in the plasma, urine and stool, as well as by genetic analysis [19, 20].

Patient history and physical examination revealed the presence of erythrodontia, skin blistering and hypopigmentation, persistent red-colored urine, as well as hemolytic anemia detected on a peripheral blood smear. The investigations confirmed the presence of excess porphyrins in the plasma. There is some correlation between genotype and phenotype, and the severity of the manifestations is usually associated with a C73R mutation, a common form of CEP [1]. There have been reports of x—linked CEP patients carrying a GATA1 mutation with misleading hematological phenotypes that include dyserythropoietic anemia, thrombocytopenia and hereditary persistence of fetal hemoglobin [21]. Other modifier genes could modulate the CEP phenotype. An example is the ALAS2 gene mutation, which is the first and rate-limiting enzyme of heme synthesis in erythroid cells [3]. Our patient had a severe form of hemolysis exhibited most of the manifestations of CEP; however, genetic analysis was not available. It is possible that the presence of this mutation caused the severe deficiency of the enzyme uroporphyrinogen III synthase and resulted in an increase in uroporphyrin I and coproporphyrin I in plasma, red cells, urine, feces, and in various tissues, explaining the presentation in our patient. However, α-thalassemia was the provisional diagnosis due to the presence of hemolytic anemia and hepatosplenomegaly in the neonatal period. This occurs in HbH disease and is a result of the deletion of three α-globin chains. The genetic test did not give us any details on the number of deletions on the alpha chain.

Alpha thalassemia is endemic to our region, and this could justify the misdiagnosis. The co-occurrence of alpha thalassemia and CEP in our patient is probably a coincidence; however, as previously reported, an interaction causing a more severe hemolytic anemia cannot be ruled out [22].

Severe cases of anemia often require frequent blood transfusions, and while this is sufficient to suppress erythropoiesis and may be effective at reducing porphyrin production and photosensitivity, it can result in iron overload and other complications [1, 23]. This can explain the improvement in the symptoms of CEP in our patient who was on frequent blood transfusions; however, once he stopped the transfusions for a period of time and was exposed to the sun, his symptoms reoccurred. Protection from sunlight exposure, minimization of skin trauma, and prompt treatment of any cutaneous infections are highly important in managing CEP. Sunscreen lotions and beta-carotene are sometimes beneficial. Concurrent desferrioxamine treatment to reduce iron overload, and hydroxyurea to suppress erythropoiesis, may provide additional benefits. While splenectomy reduces hemolysis and transfusion requirements in some patients, and while oral charcoal may increase fecal loss of porphyrins, both may be of little benefit in more severe cases [1]. A recent effort to rescue the common UROS mutation (C73R) with a pharmacological chaperone and/or a protease inhibitor has been reported [24]. At this time, the most effective treatment is a bone marrow or stem cell transplantation in early childhood, which markedly reduces porphyrin and photosensitivity levels and increases long term survival odds [1, 11, 25]. Bone marrow transplantation was not performed for our patient because of its unavailability in our country.

## Conclusion

Alpha thalassemia is a common cause of neonatal hemolytic anemia, and CEP is a rare genetic disease. Both manifest with severe hemolytic anemia, hepatosplenomegaly, and require frequent blood transfusions. The early diagnosis of alpha thalassemia in our patient could have been a factor in the delayed diagnosis of CEP, but persistent red urine, post-sunlight hypopigmented atrophic skin and erythrodontia should have been clues leading to an earlier diagnosis of CEP.

## Abbreviations

CEP: congenital erythropoietic porphyria
MPS: mucopolysaccharidosis
BCM: below costal margin
HEP: hepato-erythropoietic porphyria
UROS: uroporphyrinogen synthase
